# Online Help-Seeking Among Youth Victims of Sexual Violence Before and During COVID-19 (2016-2021): Analysis of Hotline Use Trends

**DOI:** 10.2196/44760

**Published:** 2023-08-11

**Authors:** Kimberly L Goodman, Kristyn Kamke, Tara M Mullin

**Affiliations:** 1 Rape, Abuse, and Incest National Network Washington, DC United States

**Keywords:** child sexual abuse, hotlines, COVID-19, help-seeking, online services, child abuse, mental health well-being, child support, sexual abuse, mental health service, sexual violence

## Abstract

**Background:**

Three years since the onset of COVID-19, pandemic-related trends in child sexual abuse (CSA) remain poorly understood. Common administrative surveillance metrics may have underestimated abuse during the pandemic, given youths’ limited access to mandatory reporters. Research using anonymous service-use data showed increased violence-related online help-seeking but overlooked youth-specific help-seeking for CSA during COVID-19. Understanding pandemic-related trends in CSA can inform abuse detection practices and mental health service provision for youth victims.

**Objective:**

The purpose of this study was to harness anonymous help-seeking data from the National Sexual Assault Online Hotline (NSAOH) to glean insights about CSA occurrence in the United States during the COVID-19 pandemic.

**Methods:**

We used an archival sample of victims who contacted NSAOH from 2016 to 2021 (n=41,561). We examined differences in the proportion of youth and adult victims contacting NSAOH during the first COVID-19 year (March 2020 to February 2021) compared to the prior year (March 2019 to February 2020; n=11,719). Further, we compared key characteristics of hotline interactions among youth victims during the first COVID-19 year to the prior year (n=5913). Using joinpoint regression analysis, we examined linear trends in the number of monthly sampled youth and adult victims (excluding victims of unknown age) from 2016 to 2021 who discussed any victimization event (n=26,904) and who discussed recent events (ie, events occurring during the pandemic; n=9932).

**Results:**

Most youth victims were abused by family members prior to (1013/1677, 60.4%) and after (2658/3661, 72.6%) the onset of COVID-19. The number of youth victims contacting NSAOH spiked in March 2020 and peaked in November 2020 for all youth (slope=28.2, 95% CI 18.7-37.7) and those discussing recent events (slope=17.4, 95% CI 11.1-23.6). We observed a decline in youth victims into spring 2021 for all youth (slope=–56.9, 95% CI –91.4 to –22.3) and those discussing recent events (slope=–33.7, 95% 47.3 to -20.0). The number of adult victims discussing any victimization event increased steadily from January 2018 through May 2021 (slope=3.6; 95% CI 2.9-4.2) and then declined (slope=–13.8, 95% CI –22.8 to –4.7). Trends were stable for adults discussing recent events.

**Conclusions:**

This study extends the use of hotline data to understand the implications of the pandemic on CSA. We observed increased youth help-seeking through the NSAOH coinciding with the onset of COVID-19. Trends persisted when limiting analyses to recent victimization events, suggesting increased help-seeking reflected increased CSA during COVID-19. These findings underscore the utility of anonymous online services for youth currently experiencing abuse. Further, the findings support calls for increased youth mental health services and efforts to incorporate online chat into youth-targeted services.

## Introduction

As we approach a postpandemic world, understanding trends in child maltreatment during COVID-19 remains vital for informing practices to detect abuse and ameliorate the effects of trauma. Evidence of pandemic-related trends in child maltreatment is conflicting, resulting in part from limitations of traditional surveillance strategies. Apparent declines in child maltreatment reflected in emergency room visits [1] and official reports [2] may be attributed to avoidance of emergency rooms [3] and decreased access to mandatory reporters [4] during the pandemic. These shortcomings underscore the importance of examining child maltreatment during COVID-19 with consideration of youths’ context and perspectives [5,6].

While youths’ perspectives are often assessed through self-report survey methodologies, indicators of online help-seeking are potentially valuable. Prior to the pandemic, youth actively sought help for experiences of abuse via online and text-based hotlines [7,8], which they prefer over and perceive as more private than telephone-based hotlines [8,9]. Online services offer enhanced privacy, autonomy over disclosure, and easy and immediate support, which make them acceptable and accessible for youth experiencing crisis [10], especially during a pandemic.

Recent work has explored trends in violence-related online help-seeking during the pandemic. Google searches related to child maltreatment increased during the pandemic [11,12] but may not represent help-seeking by victims. Similarly, unsupervised learning of tweets revealed how family violence was discussed during the pandemic but could not delineate whether the source was victims, perpetrators, or organizations [13]. Online help-seeking via victim-centered resources, such as online hotlines (or helplines), offer an important complementary metric.

A study of multinational child helplines [14] lacked consensus as to the presence and direction of trends in violence-related contacts early in the pandemic but did not delineate phone-based from online- or text-based hotlines. Notably, outreach to ChildHelp increased for text-based but not phone-based contacts early in the pandemic [15]. While these studies demonstrated the promise of hotline data for understanding child maltreatment during the pandemic, their limited focus on overall hotline utilization conflated victim and nonvictim contacts. Research examining hotline use specific to youth victims during the pandemic is needed.

Amid these challenges, research on pandemic-related child maltreatment has largely overlooked child sexual abuse (CSA). However, concerns about victims’ physical and social isolation and accessibility to perpetrators during stay-at-home orders [16] are particularly applicable to CSA [17]. To address this gap in understanding of pandemic-related trends in CSA, we examine archival data from the National Sexual Assault Online Hotline (NSAOH).

We advance knowledge by addressing limitations of previous work on 3 fronts. First, we examine online help-seeking for sexual violence among youth victims specifically. Second, we incorporate 5 years of observations (2016-2021), providing a broader picture of help-seeking patterns across the pre- and postpandemic onset time periods. Third, we delineate recent events to distinguish violence that occurred during the pandemic.

## Methods

### Data Collection

The NSAOH is a US-based online hotline providing 24/7 anonymous crisis intervention services via desktop and mobile internet chat. RAINN (Rape, Abuse & Incest National Network), an anti–sexual violence organization in the United States, created and has operated NSAOH since 2006 to serve victims of sexual violence.

Data collection via an in-depth online assessment (called the “session assessment”) serves an integral role in hotline operations to identify areas for service improvement and staff training. Directly following the first chat session of their shift, staff record session information, such as event characteristics and topics discussed. Because information is not requested for the purpose of completing the assessment, data are often considered unknown (ie, missing). Staff receive instructions for assessment completion both within the assessment and through supplemental training.

Data in this study represent a sample of all hotline chats in which the visitor discussed an experience of sexual or other interpersonal violence (ie, victims) between January 2016 and December 2021 (N=41,561). Additional information about hotline procedures, the organizational context, and sample inclusion criteria are available in Multimedia Appendix 1.

### Ethical Considerations

Data used in this study were originally collected as part of ongoing internal program evaluation; thus, informed consent was not required. Analysis of this archival data for the purposes of generating generalizable knowledge of online hotline users and sexual violence experiences was deemed exempt by the Advarra institutional review board under category 4 of the Revised Common Rule.

### Statistical Analysis

We examined differences in the proportion of youth (ie, those younger than 18 years) and adult (aged 18 years and older) victims during the first COVID-19 year (March 2020 through February 2021) compared to the year prior (March 2019 through February 2020; n=11,719), with victims of unknown age excluded (5178/16,897, 31%). Among youth (n=5913), we compared perpetrator type (family vs nonfamily), perpetrator living status (currently living with vs not currently living with the victim), event timeframe (whether the event occurred within the last month, 1 month to 1 year ago, or over 1 year ago), and event frequency (repeated abuse vs single occurrence) during the first COVID-19 year compared to the year prior. These analyses used the chi-squared test with pairwise exclusion of missing data; analyses used SPSS (version 28.0; IBM Corp).

To contextualize trends in youth help-seeking, we used Joinpoint (version 4.9.1.0; National Cancer Institute) to examine linear trends in the number of monthly sampled victims by age group from 2016 to 2021 among all youth and adult victims (n=26,904; Multimedia Appendix 1 provides additional information about joinpoint analyses). Cases of unknown age were excluded (n=14,657; 22.7%-50.3% missingness per month). We repeated this analysis among youth and adult victims known to have discussed recent events that occurred within a month of contacting the hotline (n=9932). We also graphed the proportion of monthly sampled youth victims by year among all victims and those discussing recent events. Data were analyzed in spring 2022.

## Results

### Sample Characteristics

We observed a significant increase in the proportion of youth victims during the first COVID-19 year relative to the year prior (1871/4560, 41% vs 4042/7159, 56.5%). Among youth, we noted statistically significant but small shifts in the proportion of chats that involved family-perpetrated assaults (1013/1677, 60.4% vs 2658/3661, 72.6%), perpetrators currently living with the victim (1028/1733, 59.3% vs 2815/3841, 73.3%), recent assaults (ie, within the last month; 1017/1313, 77.5% vs 2381/2774, 85.8%), and repeated assaults (1397/1764, 79.2% vs 3250/3773, 86.1%). Table 1 shows full sample characteristics and missingness, as well as characteristics and comparisons using pairwise deletion.

**Table 1:** Comparison of victim and event characteristics in the pre-COVID year (March 1, 2019-February 29, 2020) and the COVID year (March 1, 2020-February 28, 2021). Information about response option coding for table categories is available in Multimedia Appendix 1.

				Full sample (n=16,897)			Pairwise deletion (n=11,719)									
				Pre-COVID year (n=7139), n (%)	COVID year (n=9758), n (%)	Pre-COVID year (n=4560), n (%)		COVID year (n=7159), n (%)			Effect size (Cramér's V)^a^				*P* value^a^	
**Victim age group**												0.15				<.001
		Youth		1871 (26.2)	4042 (41.4)	1871 (41)		4042 (56.5)								
		Adult		2689 (37.7)	3117 (31.9)	2689 (59)		3117 (43.5)								
		Unknown		2579 (36.1)	2599 (26.6)	N/A^b^		N/A								
**Characteristics among youth (n=5913)**																
	**Perpetrator**									0.12				<.001		
			Family member	1013 (54.1)	2658 (65.8)	1013 (60.4)		2658 (72.6)								
			Non–family member	664 (35.5)	1003 (24.8)	664 (39.6)		1003 (27.4)								
			Unknown	194 (10.4)	381 (9.4)	N/A		N/A								
	**Victim living with perpetrator**									0.14				<.001		
			Yes, currently^c^	1028 (54.9)	2815 (69.6)	1028 (59.3)		2815 (73.3)								
			Not currently^c^	705 (37.7)	1026 (25.4)	705 (40.7)		1026 (26.7)								
			Unknown	138 (7.4)	201 (5)	N/A		N/A								
	**Event timeframe**									0.10				<.001		
			Within the last month	1017 (54.4)	2381 (58.9)	1017 (77.5)		2381 (85.8)								
			1 month to 1 year ago	132 (7.1)	183 (4.5)	132 (10.1)		183 (6.6)								
			Over a year ago	164 (8.8)	210 (5.2)	164 (12.5)		210 (7.6)								
			Unknown	558 (29.8)	1268 (31.4)	N/A		N/A								
	**Event frequency**									0.09				<.001		
			Repeated	1397 (74.7)	3250 (80.4)	1397 (79.2)		3250 (86.1)								
			Single occurrence	367 (19.6)	523 (12.9)	367 (20.8)		523 (13.9)								
			Unknown	107 (5.7)	269 (6.7)	N/A		N/A								

^a^Chi-square comparisons; unknown (“missing”) data were excluded in these comparisons.

^b^N/A: not applicable.

^b^“Currently” means at the time of contacting the hotline.

### Joinpoint Regression Analysis

**Figure 1:**
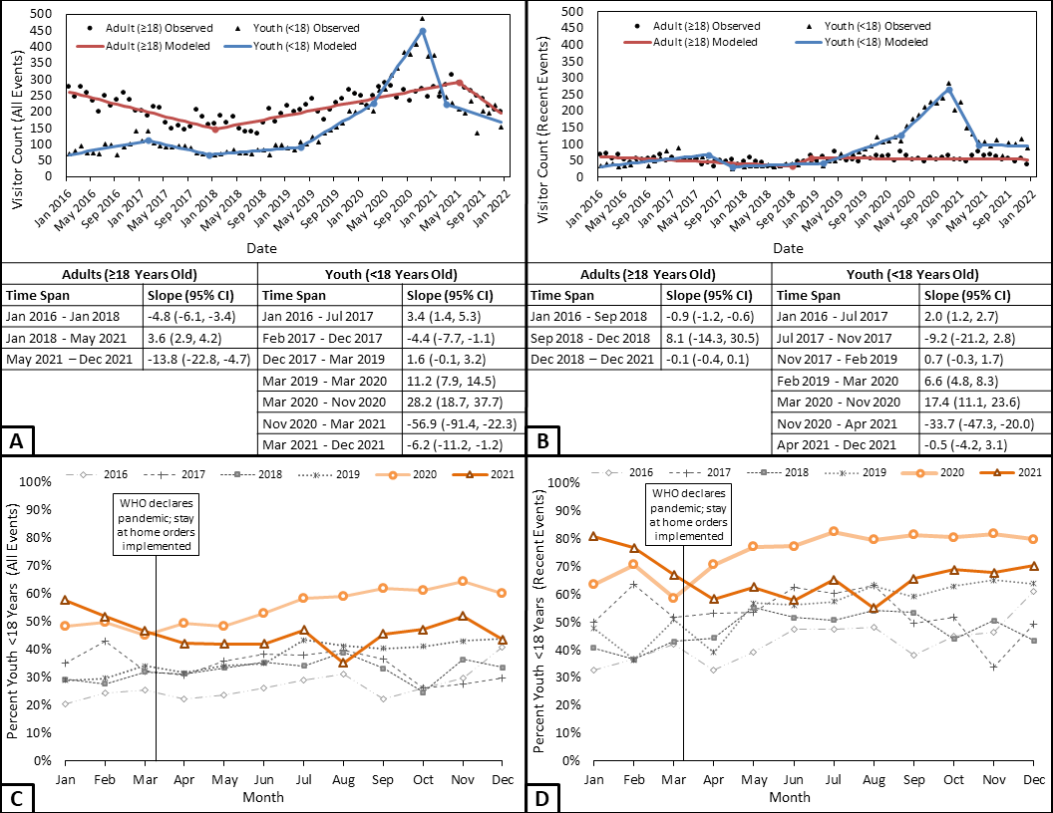
Linear trends in National Sexual Assault Online Hotline use among youth. Analyses were limited to hotline visitors who identified as victims, with the sample reflecting the first chat of a staff member's shift. Panels A and C display all events, whereas panels B and D reflect chats discussing recent events (ie, occurring within the last month). Data are from January 2016 to December 2021 and were analyzed using Joinpoint (version 4.9.1.0; National Cancer Institute). WHO: World Health Organization.

Inflection points reveal variability in the strength or direction (or both) of trends in the number of victims by age in our sample (Figure 1). Prior to 2019, the number of youth victims fluctuated. In the year prior to COVID onset, the number of all youth victims and those discussing recent events increased by an average of 11.2 (95% CI 7.9-14.5) and 6.6 (95% CI 4.8, 8.3) per month, respectively. The most striking trend commenced in March 2020 at the onset of the pandemic: there were average monthly increases of 28.2 (95% CI 18.7-37.7) and 17.4 (95% CI 11.1-23.6) for all youth victims and youth discussing recent events, respectively. Both the number and proportion of youth victims peaked in November 2020. Subsequently, youth victims declined into early spring 2021, both overall (slope=–56.9; 95% CI –91.4 to –22.3) and for recent events (slope=–33.7; 95% CI –47.3 to –20.0). We observed an increase in all adult victims starting in January 2018 (slope=3.6; 95% CI 2.9-4.2), but this trend was consistent through May 2021, after which adult victims declined into December 2021 (slope=–13.8, 95% CI –22.8 to –4.7). The number of adult victims discussing recent events was largely consistent from 2016 to 2021.

## Discussion

### Principal Findings

This study is the first, to our knowledge, to reveal increases in youth help-seeking for sexual violence coinciding with the onset of COVID-19. These increases peaked in November 2020 and were present for all youth victims and for youth victims discussing recent events. Although the number of adult victims increased, this trend was more gradual, preceded the onset of COVID-19, and was not present for those discussing recent events. Our finding that trends were sustained for recent events suggests an increase in youth affected by sexual violence during the pandemic rather than increased help-seeking among all victims for current and past events due to limited alternative support options.

While our findings seemingly conflict with declines in abuse-related hospital visits and official reports [1,2], evidence of pandemic-related trends in child maltreatment must be interpreted in light of the pandemic context [6]. Stay-at-home orders limited youths’ access to formal services and mandated reporters, but online hotlines remained accessible to youth victims seeking help. Other studies used overall hotline utilization data to estimate pandemic-related child maltreatment [14,15] but conflated help-seeking among youth victims with that of adult victims and other hotline users (eg, supporters, general information seekers, or prank visitors) whose hotline use may have been differentially impacted by COVID-19. We also examined help-seeking for recent victimization, strengthening inferences that increased hotline contacts reflected help-seeking for violence experienced during the pandemic rather than past victimization. These procedures provide a model for how anonymous online hotline data can be used as a surveillance indicator for child maltreatment.

Most youth who contacted the NSAOH—both prior to and during the pandemic—discussed repeated experiences of violence perpetrated by family members or someone else living with them. During the pandemic, we observed small proportional increases in youths’ chats that featured these characteristics, consistent with elevated violence driven by victims’ increased accessibility to perpetrators in the home amid limited contact with trusted adults (eg, teachers). Future work should examine to what extent increased youth help-seeking reflected increased frequency and severity of ongoing violence predating the pandemic or cases of violence that began during the pandemic.

The number of chats from youth began to decrease after November 2020. This decline could relate to youth having a greater ability to avoid perpetrators or opportunities to disclose to alternative sources of support as schools reopened in the summer and fall of 2020. However, because reopening policies were variable across the United States, driven by state and local government, this trend should be interpreted with caution.

While reported trends were transitory, the effects of sexual abuse are not. Our findings reflect increased help-seeking by youth during the pandemic, an indication of rising demand for mental health services to treat trauma and its sequelae among youth. Given the national shortage of child mental health providers that predates COVID-19 [18], there is a pressing need to expand youth services to address the complex trauma resulting from CSA and other effects of COVID-19 [4].

### Limitations

Joinpoint regression analysis allowed us to identify changes in the linear trend of victims’ hotline use from 2016 to 2021. While we interpreted the reason for some of these changes, their causes cannot be definitively ascertained from our analysis. We could not differentiate repeat hotline users from new users, which would help delineate new cases of abuse. Our data collection protocol prioritized the delivery of victim-centered services. We did not solicit information from visitors for assessment purposes, resulting in some missing data and limited demographic information. Future work could incorporate anonymous collection of demographics prior to service provision to enhance the utility of hotline data as a surveillance indicator of child maltreatment. Understanding the interplay of hotline supply and demand will also be useful because changes in staff availability (ie, supply) could affect the accessibility and use of services.

### Conclusions

Our study demonstrates the utility of anonymous online hotline data as a complementary surveillance indicator for child maltreatment that is transferrable to other public health priority areas (eg, mental health). Increased help-seeking amid decreased safety at home also speaks to the utility of online support options for youth experiencing abuse. Preparing for future public health emergencies necessitates consideration of additional online communication options that allow youth to privately disclose abuse to trusted adults. Future work should explore how to safely incorporate these communication options into existing services, such as online schooling or telemedicine platforms.

## Appendix

Multimedia Appendix 1 Expanded description of study methodology.

## Abbreviations

CSA: child sexual abuse
NSAOH: National Sexual Assault Online Hotline
RAINN: Rape, Abuse & Incest National Network

## Data Availability

The data sets generated and analyzed during this study are not publicly available due to the sensitivity of the data but aggregate data used in joinpoint analyses are available from the corresponding author on reasonable request.
