# Supplementary material for: Online Help-Seeking Among Youth Victims of Sexual Violence Before and During COVID-19 (2016-2021): Analysis of Hotline Use Trends
Source: JMIR Public Health Surveill. 2023 Aug 11;9:e44760. doi: 10.2196/44760 (PMC10457693; doi:10.2196/44760)
Supplement: Multimedia Appendix 1 [file publichealth_v9i1e44760_app1.docx]

Appendix: Expanded Description of Study Methodology

# Overview of National Sexual Assault Online Hotline

The National Sexual Assault Online Hotline (NSAOH) provides 24/7 chat support to individuals affected by interpersonal (primarily sexual) violence, including survivors, family members and friends of survivors, and service providers. Additionally, visitors include information seekers, off-target and prank calls, perpetrators or potential perpetrators, and visitors who never spoke or did not provide enough information to be classified.

The NSAOH was created and is operated by the Rape, Abuse & Incest National Network (RAINN). The National Sexual Assault Hotline (NSAH) commonly refers to RAINN’s online and telephone hotlines jointly. Importantly, the online hotline (NSAOH) is distinct because it is operated by RAINN employees and volunteers, whereas the telephone hotline is primarily administered by local sexual assault service providers. The NSAOH is available to internet users via RAINN’s website or mobile app. To join a chat, users must agree to the NSAOH Terms of Service. After agreeing, they are taken to a virtual waiting room until a support specialist (i.e., hotline staff or volunteer) can begin their private online chat.

# Current Study Methodology

The survey instrument (“Session Assessment”) described in this paper was initially developed for internal hotline operations purposes and has historically aided in the identification of areas for service improvement and staff training. As part of their roles on the Research & Evaluation team at RAINN, the authors developed the Session Assessment, manage and update the assessment in Survey Monkey, analyze assessment data, and disseminate findings internally. Further, they conduct independently conceptualized research using this data to inform general knowledge, policy, and practice.

The Session Assessment begins with questions about the **visitor** (i.e., any person who connects to the online hotline). Later in the assessment, if a violent event is discussed, staff indicate whether the visitor was the victim of the event discussed. To reduce staff burden while retaining a representative sample of victims, staff (i.e., paid staff and volunteers) complete the assessment once per shift, following their first chat session. The Terms of Service informs visitors that non-identifying information about their chats may be used and shared in aggregate form to better understand the needs of sexual assault survivors.

Our team routinely updates the Session Assessment in response to staff feedback, current areas of interest, and updated guidelines on collecting sensitive information. These revisions include adding, removing, or revising questions and response options. Below, the format of the questions in the most recent assessment version (for the study period) is displayed.

# Study Inclusion/Exclusion Variables

- Included cases were those in which:
  - The user accessed the online hotline between January 2016 and December 2021
  - The assessment was completed for the first session of the staffer’s shift
  - The assessment was completed for all required questions
  - The user did not explicitly disclose being outside of the United States
  - A violent event was discussed
  - The user identified as the victim of the event.

Specifically, the following assessment questions (written in italics) and bolded responses indicate inclusion criteria:

## Visitor Location

- *Was the visitor accessing the OHL [NSAOH] from outside the United States?*
  - Yes
  - **No**
  - **Unknown**

**Note:** Although NSAOH is a US-based hotline, we sometimes receive users from outside the United States. However, the users’ location is unknown unless the visitor explicitly discloses it. Thus, staff frequently select “Unknown” for the above question. Thus, we excluded users who were known to be accessing the NSAOH from outside the US (<5%) but retained visitors whose location was “Unknown” in addition to users who were within the US.

## Event discussed

- W*as an event/incident relating to sexual or physical violence discussed?*
  - No, issues discussed did not pertain to sexual or physical violence, or related incidents
  - No, visitor contacted OHL [NSAOH] for general information only
  - **Yes, an event/incident was discussed relating to sexual assault or domestic violence**

**Note:** Some victims do not explicitly disclose the type of event they experienced during their chat, although they may have experienced sexual violence. In particular, youth may not have the language to describe a sexual assault. Additionally, some victims emphasize interpersonal violence events outside the scope of sexual violence (~3%), although sexual violence may also be discussed. Thus, all visitors who identified as victims were retained in analyses.

## Visitor Type

- *Who was the visitor in relation to the victim of the event?*
  - **Visitor was the victim**
  - Intimate Partner/Spouse of the victim
  - Other family member of the victim
  - Friend/Acquaintance of the victim
  - Allied professional (victim advocate, social worker, attorney, medical or mental health care provider)
  - Perpetrator
  - Undisclosed
  - None of the above/Other (please explain why response does not fit in categories above)

# CHARACTERISTICS OF VICTIMS

## Visitor Age

We used the victim’s **current** age in descriptive and trend analyses. To determine the visitor’s current age, we used the question below:

- *Was the visitor an adult or minor at the time of the session?*
  - Adult
  - Minor (under 18 years of age)
  - Unknown

Sometimes visitors explicitly disclose their age, but often, staff use clues from their chats with visitors to report whether the visitor is an adult or minor. Listed below are examples of clues used to identify the visitor as an adult:

- Visitor provided timeframe of event clues (e.g., “10 years ago in middle school”, “a few years ago in college”)
- Visitor discussed their ‘career’ (but not ‘work’ generally)
- Visitor discussed being in college generally (not ‘early college’) [but not a Freshmen in college who could be younger than 18]
- Visitor discussed being in graduate school
- Visitor discussed legal matters, such as divorce, buying / selling a house, etc.

The following are examples of clues used to identify the visitor as a minor:

- Visitor used indirect age identifiers (“other kids,” “I’m a bad kid,” “I can’t because I’m not old enough,” distinguishing between self and adults)
- Visitor was in Elementary or Middle School
- Visitor was a Freshman or Sophomore in high school [but not a Junior or Senior who could be 18 or older]
- Visitor discussed legal matters, such as foster care, guardians, mandated reporting, etc.
- Visitor discussed kid-specific topics (has a “babysitter,” “playdate,” “sleepover,” “recess”)
- Visitor referenced puberty (e.g., starting to menstruate)
- Visitor requested child-specific resources/services if they were the victim

After determining the visitor is the **victim** of the event discussed (see above), staff indicate the age of the victim at the time of the event, shown below:

- *Was the victim a minor or an adult at the time of the event?*
  - Minor (under 18 years of age)
  - Adult
  - Unknown

Staff answer this question based on the victim’s disclosure of their age at the time of the assault or their current age and how long it has been since they were assaulted.

To create the age characteristic used in this study, we examined discrepancies in responses to the two questions. Discrepancies occurred in 1% of cases. These included cases in which staff indicated that the visitor was a minor at the time of the chat and an adult at the time of the event, the visitor was a minor at the time of the chat but an unknown age at the time of the event, and the visitor’s age was unknown at the time of the chat but an adult at the time of the event. Discrepant responses were recoded as “Unknown.” Otherwise, we used the victim’s age at the time of the contact.

## Event Timeframe

This study used a recoded version of our timeframe variable in descriptive and trend analyses. Below, we include the assessment question (italicized), response options (left), and how it was recoded for analyses (right).

- *What was the approximate timeframe of the event? (If the event has occurred repeatedly, please indicate when the most recent event occurred.)*

| - - < 3 hours ago   - 3-12 hours ago   - 12-24 hours ago   - 1-7 days ago   - Within last month | Within the last month |
| --- | --- |
| - - Within 6 months   - Within year | 1 month – 1 year ago |
| - - 1-5 years   - >5 years | Over a year ago |
| - - Undisclosed | Unknown |

## Victim-Perpetrator Relationship

This study used a recoded version of our victim-perpetrator relationship variable in descriptive analyses. Below, we include the assessment question (italicized), response options (left), and how it was recoded for analyses (right).

- *To the best of your ability please indicate the perpetrator’s relationship with the victim at the time of the event.  The perpetrator was a(n) ________ of the victim.*

| - - Family Member (other than spouse; can include step-family and foster family) | Family member |
| --- | --- |
| - - Acquaintance (e.g., date, neighbors, etc.)   - Authority Figure (e.g., coach, religious leader, professor, boss, babysitter, *not* trafficker)   - Coworker/Colleague/Classmate   - Client/Person Purchasing Sex   - Friend   - Intimate Partner/Spouse (current or ex; can include dating partner, boyfriend/girlfriend, husband/wife, ongoing sexual partner)   - Medical or Service Provider (doctors, nurses, massage therapists, mental health professionals)   - Partner/Friend of a Family Member (mom’s boyfriend, brother’s friend, godfather)   - Person Briefly Known (e.g., someone just met, blind date, someone met at a party/bar)   - Stranger   - Trafficker | Non-family |
| - - Undisclosed | Unknown |
| - - None of the Above, Please Specify Relationship (open-ended comment box provided) | Open-ended responses were recoded into one of the closed response options. Answers that could not be recoded into one of these response options were considered Unknown. |

**Note:** Some victims discussed events involving multiple perpetrators. Prior to 11/25/2020, when the event discussed involved multiple perpetrators, staff were instructed to describe the perpetrator’s relationship to the victim in the None of the Above/Other open-ended comment box. These responses were recoded to select the victim-perpetrator relationship category that described the perpetrator with the closest relationship to the victim. We considered “family member” to be the closest relationship, so any multiple perpetrator response describing one or more family members was recoded as such, while other multiple perpetrator responses were coded as “Non-family.”

On 11/25/2020, our assessment was updated to collect more detail about events involving multiple perpetrators. Following this change, the assessment allowed for several characteristics, including the victim-perpetrator relationship, to be collected for up to 5 perpetrators involved in multiple perpetrator events. For multiple perpetrator cases after this change was made, the victim-perpetrator relationship for events involving one or more family members was recoded as “Family member.” If none of the perpetrators were family members, but at least one perpetrator was known to be non-family (consistent with the coding above), the victim-perpetrator relationship was recoded as “Non-family.” If all victim-perpetrator relationships were unknown/undisclosed, the victim-perpetrator relationship was considered “Unknown.”

## Victim Currently Living with Perpetrator

- *Is the perpetrator living with the victim currently?*
  - Yes, currently
  - Not currently
  - Unknown

**Note:** Some victims discussed events involving multiple perpetrators. On 11/25/2020, after the assessment was updated, living with the perpetrator was collected for up to 5 perpetrators involved in the event. For cases where at least one perpetrator was currently living with the victim, the case was coded as “currently living with the victim.” If none of the perpetrators were currently living with the victim and at least one was known to *not* be living with the victim, the case was coded as “not currently living with the victim.” If, for all perpetrators, it was unknown/undisclosed whether the victim was currently living with the perpetrator, cases were coded as Unknown.

## Event Frequency

- *What was the frequency of the assault?*

| - - Repeated, still occurring   - Repeated, no longer occurring | Repeated |
| --- | --- |
| - - One time | Single occurrence |
| - - Undisclosed | Unknown |

# Additional Information on time trend analyses

This study utilized Joinpoint Regression statistical software version 4.9.1.0 for time trends analyses (<https://surveillance.cancer.gov/joinpoint>/). Joinpoint was developed by the Surveillance Research Program at the Nation Cancer Institute.

The software allows the user to fit data with “joinpoints” that indicate the start/end of a new trend line, allowing for the identification of multiple changes in trends. Users can also test whether trends are statistically significant. The software starts with 0 joinpoints (e.g., a straight line). Then, it tests whether additional joinpoints are statistically significant and must be added to the model (up to the user set maximum number). We tested for 0-7 joinpoints in our models, specifying a minimum of 2 data points between joinpoints and 2 data points between each end of the data and a joinpoint. Because we used count data in our joinpoint analyses, we tested a linear model with errors modeled using Poisson variance. We used the BIC3 model to select the best fitting model. The BIC3 model is considered the closest estimate of the computationally intensive Monte Carlo Permutation test originally proposed to perform joinpoint regression analyses.^^[[1]](#footnote-1)^^

1. Kim HJ, Fay MP, Feuer EJ, Midthune DN. Permutation tests for joinpoint regression with applications to cancer rates. Stat Med 2000;19:335-51 (correction: 2001;20:655). doi: 10.1002/(sici)1097-0258(20000215)19:3<335::aid-sim336>3.0.co;2-z [↑](#footnote-ref-1)
